# Solvent-Free Process for the Development of Photocatalytic Membranes

**DOI:** 10.3390/molecules24244481

**Published:** 2019-12-06

**Authors:** Rosa M. Huertas, Maria C. Fraga, João G. Crespo, Vanessa J. Pereira

**Affiliations:** 1 iBET, Instituto de Biologia Experimental e Tecnológica, Apartado 12, 2780-901 Oeiras, Portugal; rosa.huertas@ibet.pt (R.M.H.); mariadocarmo.fraga@gmail.com (M.C.F.); 2 LAQV-REQUIMTE, Departamento de Química, Faculdade de Ciências e Tecnologia, Universidade Nova de Lisboa (UNL), 2829-516 Caparica, Portugal; jgc@fct.unl.pt; 3 Instituto de Tecnologia Química e Biológica António Xavier, Universidade Nova de Lisboa, 2784 Oeiras, Portugal

**Keywords:** photocatalytic membranes, solvent-free sol-gel modification, temperature effect, membrane morphology, photocatalytic performance

## Abstract

This work described a new sustainable method for the fabrication of ceramic membranes with high photocatalytic activity, through a simple sol-gel route. The photocatalytic surfaces, prepared at low temperature and under solvent-free conditions, exhibited a narrow pore size distribution and homogeneity without cracks. These surfaces have shown a highly efficient and reproducible behavior for the degradation of methylene blue. Given their characterization results, the microfiltration photocatalytic membranes produced in this study using solvent-free conditions are expected to effectively retain microorganisms, such as bacteria and fungi that could then be inactivated by photocatalysis.

## 1. Introduction

Silicon carbide membranes have been reported as effective supports that can be easily functionalized to provide materials with catalytic activity [1,2]. Moreover, due to its chemical and thermal resistance as well as mechanical properties, they are useful for harsh environmental and sustainable process applications [3,4,5]. Previous works demonstrated that silicon carbide membranes have a smooth top layer, controlled porosity, high permeability, and proved to be efficient in removing total suspended solids, as well as oil and grease when used to treat oily wastewaters [4,6]. The main problem associated with the use of membranes is fouling formation that can decrease the flux and the lifetime of the membrane.

Photocatalytic membrane reactors that combine filtration and degradation/inactivation in the same compartment have been described to have great potential for use in water and wastewater treatment [7]. Combining advanced oxidation processes with membrane filtration can overcome fouling problems and increase the effectiveness of the treatment process. The modification of membranes can also be used to decrease their molecular weight cut-off by reducing the pore size.

Titanium dioxide (TiO_2_) is the most commonly used material for the fabrication of photocatalytic membranes due to its high reactivity, chemical and thermal stability, low cost, reusability, and high photocatalytic performance [7,8,9].

Sol-gel has been widely reported as effective for the synthesis of TiO_2_-based materials [10,11]. This technique can be used to modify different supports, such as membranes, to produce photocatalytic membranes, control their porosity [12,13,14,15], and eliminate surface defects.

The combination of silicon dioxide (SiO_2_) and TiO_2_ has been reported to improve the photocatalytic activity of TiO_2_ [16,17,18].

In previous work, a sol-gel process using ethanol as a solvent was used to coat SiC membranes with silicon dioxide (SiO_2_) and TiO_2_ Degussa nanoparticles [19]. As a result, reusable photocatalytic membranes were obtained with a lower molecular weight cut-off, as well as higher hydrophilicity and oleophilicity, compared to the unmodified membranes. Assays performed in a dead-end filtration system combined with UV light confirmed their high potential to degrade organic contaminants [19].

Extremely high removals of total suspended solids, chemical oxygen demand, total organic carbon, phenolic and volatile compounds were obtained when these hybrid photocatalytic membranes were tested in a new hybrid photocatalytic membrane reactor to treat olive mill wastewaters [20].

Since the sol-gel procedure employs a high volume of solvent to modify large membrane areas needed at industrial scale, the development of solvent-free modification methods directly bound up with the principle of green chemistry [21], and would therefore highly increase the sustainability besides decreasing the membrane production costs.

The aim of the present work was to validate a new low cost and environmentally friendly methodology—based on the use of aqueous solutions—to produce SiC membranes with high photocatalytic activity.

## 2. Results and Discussion

### 2.1. Photocatalytic Performance

**Figure 1:**
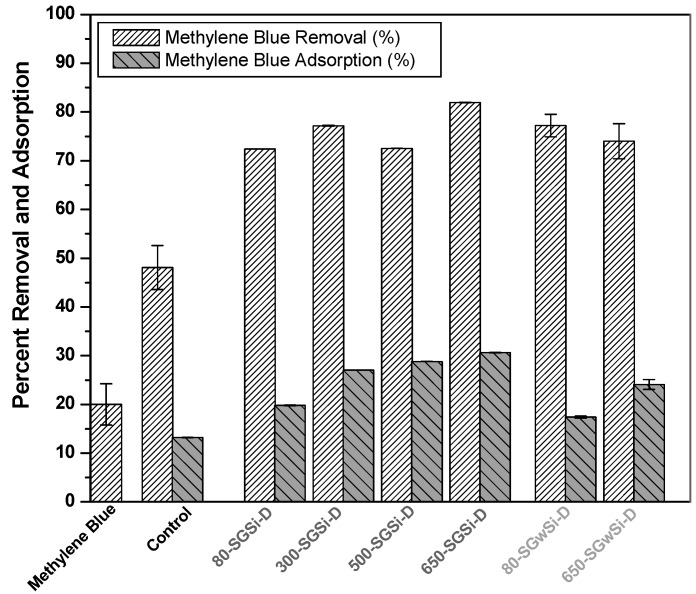
Comparison of the removal of methylene blue after 60 min (direct photolysis) with removal and adsorption of methylene blue using the control and the modified membranes.

The total percent removal (photocatalytic activity, including adsorption obtained after 60 min exposure to UV light) and the adsorption of methylene blue obtained for the different membranes are shown in Figure 1 and Table 1. The removal of methylene blue was also measured in the absence of the photocatalyst (without membranes) under the same experimental conditions (Figure 1). A single experiment was conducted to test the photocatalytic activity of the membranes modified using solvent. In these experiments, the error was extremely low because it corresponded to four measurements of methylene blue concentration obtained after each photocatalytic experiment. The higher errors observed for the other samples (methylene blue, control, and solvent-free membranes) could be explained since they were calculated taking into account two duplicate experiments. Two duplicate experiments were conducted to test the photodegradation of methylene blue, as well as the photocatalytic performance of the unmodified and modified membranes (using two different pieces of the membrane).

The increase in the direct photolysis results obtained in these experiments (Figure 1, methylene blue column) compared to previous work [19] was due to a different medium pressure ultraviolet lamp used in this work. The removal due to direct photolysis of methylene blue (without membrane) was around 20%, whereas when the control membrane was tested, an important self-photoactivity was observed, which was expected taking into account the results obtained in previous work [19], achieving a percent removal of 48%.

The modified membranes obtained were identified as T-SGwSi-D and T-SGwSi-D, where T denotes the temperature applied over modified substrates samples, and the letter w differentiates the samples prepared using water as a matrix instead of solvent in the sol-gel process. Table 1 shows that the increase in the percent removals obtained by adding the photocatalytic layers was between 24% and 34%.

The total removal obtained for modified membranes using solvent during the sol-gel process and subjected to different temperatures 80-SGSi-D, 300-SGSi-D, and 500-SGSi-D was around 76% that proved to be 1.5–1.6 times more photocatalytically active than the control membrane. Even though the membrane 650-SGSi-D subjected to the highest temperature during the thermal protocol achieved the highest total removal of 82% (Figure 1; Table 1), the differences in the photocatalytic behavior of this and the other thermally-treated membranes were not notorious (10% difference between the removal efficiency of the membrane subject to 80 °C and 650 °C). This behavior could be due to the similarities among properties that influence the photocatalytic activity, such as surface area, number of active sites for radical formation, pore size distribution, or pore accessibility.

The solvent-free modified photocatalytic membranes were, therefore, prepared using the lowest temperature (80 °C), which would lead to reduced costs in terms of energy consumption, as well as the highest temperature (650 °C), which achieved the best performance when the membranes were modified with solvent. Distilled water was used as an aqueous solution for the sol-gel process to produce the T-SGwSi-D membranes. The membranes obtained were named 80-SGwSi-D and 650-SGwSi-D.

Figure 1 shows that the 80-SGwSi-D membrane achieved higher total removal and lower adsorption (Table 1) than the 650-SGwSi-D membrane and, therefore, had the highest photocatalytic effectiveness (1.6 times better than the control membrane).

Pseudo-first-order decay kinetics was observed. The time-based degradation rate constant (k_t_) was obtained from the slope of the relation between ln(C_t_/C_0_) and time (Equation (1)), where C_0_ and C_t_ correspond to the concentration of the dye measured before and after different UV exposures times.

(1) ln(CtC0)=−k.t

Table 2 and Figure S1 present the time-based degradation rate constants and the half-life times determined. The degradation rate constants and Equation (1) could be used to predict how the degradation of methylene blue is expected to change as a function of the exposure time.

#### Assessment of the Reusability Potential of the Membranes

**Figure 2:**
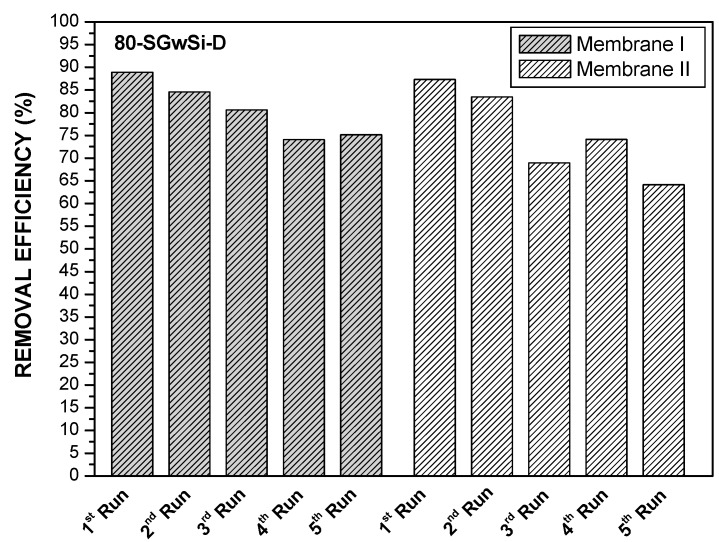

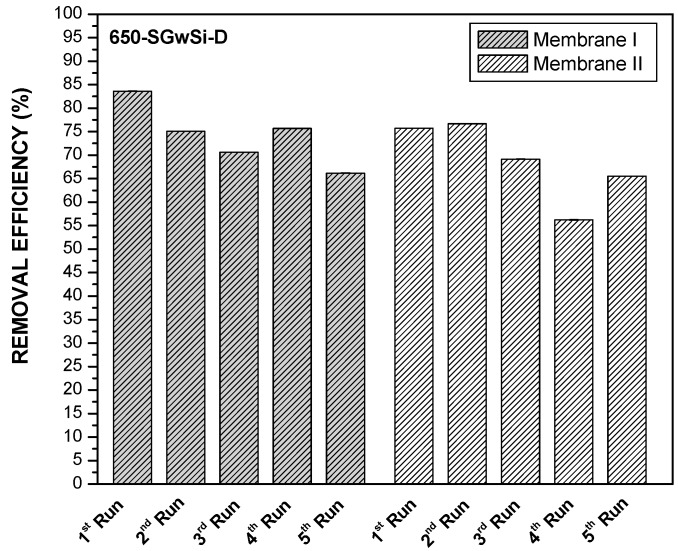
Removal efficiency obtained after 60 min for five consecutive photocatalytic experiments using two different membrane batches for the solvent-free 80-SGwSi-D (top) and 650-SGwSi-D (bottom) modified membranes.

One of the pre-requisites for using the developed photocatalytic membranes at the industrial level is to investigate the long-term stability and reusability of these materials. Thus, the photocatalytic activity of the membranes prepared (80-SGwSi-D and 650-SGwSi-D) was evaluated and compared after five consecutive degradation experiments using two different batches of silicon carbide membranes (labeled as Membrane I and Membrane II), prepared using the same chemical and thermal protocols. The results obtained under the same experimental conditions are depicted in Figure 2. The error was calculated based on four measurements of methylene blue concentration obtained after each photocatalytic experiment.

When comparing the membranes (T-SGSi-D) prepared by the deposition of SiO_2_-TiO_2_ using a solvent (Figure 1) with the solvent-free process (T-SGwSi-D), we could observe that the photocatalytic activity of the solvent-free process achieved higher removal values (up to 89% for the membrane 80-SGwSi-D; Figure 2). Comparing the efficiency of the modified membranes, we could observe that the photocatalytic activity of the 80-SGwSi-D membrane was roughly maintained after five repeated runs with an average total removal of 81% ± 6 and 76% ± 10 for Membrane I and Membrane II, respectively (Figure 2), whereas the membrane 650-SGwSi-D showed lower photocatalytic effectiveness and/or higher variability (with an average total degradation of 74% ± 11 and 69% ± 11 obtained for Membrane I and Membrane II, respectively). Similar average total degradation values were obtained using the two thermal protocols tested. These results showed that using a lower temperature in the final thermal protocol was enough to induce photocatalytic activity, which would lead to energy savings.

**Figure 3:**
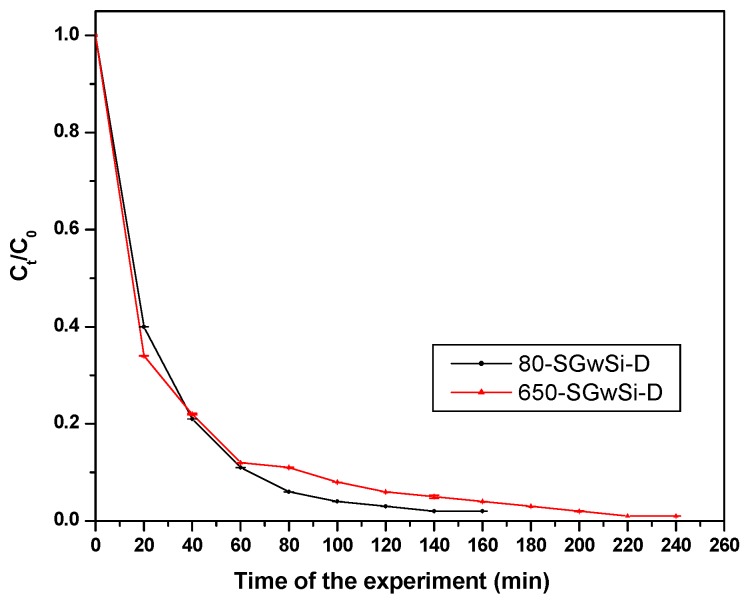
Total removal photocatalytic experiment conducted with the solvent-free membranes 80-SGwSi-D and 650-SGwSi-D.

A final long term assay was conducted using the solvent-free membranes. Removal below the detection limits of methylene blue was achieved after 240 min of UV exposure for the 650-SGwSi-D membrane and after 160 min for 80-SGwSi-D being the latter the most efficient membrane (Figure 3). Error bars correspond to duplicate experiments.

**Figure 4:**
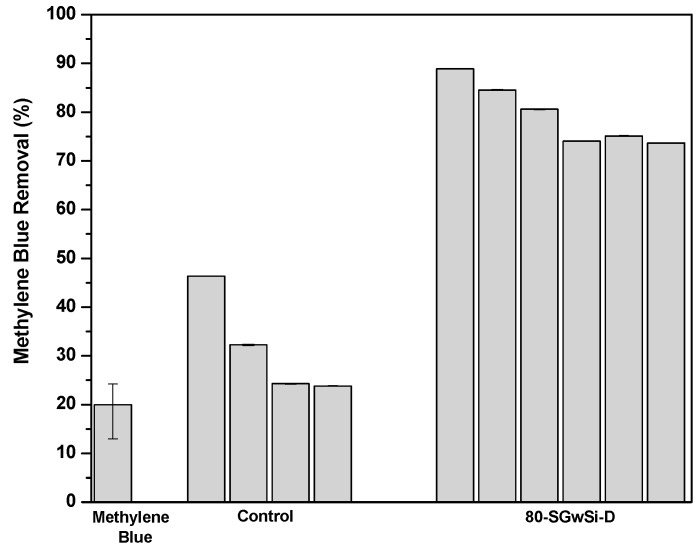
Comparison of the degradation of methylene blue by direct photolysis with the removal of methylene blue using the unmodified substrates (control) and solvent-free 80-SGSi-D modified membrane (indirect photolysis).

Comparing the efficiency of the membrane 80-SGwSi-D (Membrane I) with the control membrane, we could observe a stabilization of the photocatalytic performance around 75%, maintaining a good performance after repeated six runs. On the contrary, the photo corrosion of the silicon carbide material [22] of the control membrane could induce a high decrease in the removal performance observed after three runs, achieving a removal efficiency up to 24% in the third run, which was extremely similar to the removal of methylene blue by direct photolysis (Figure 4).

In these consecutive experiments, the error obtained for the control and the modified membrane was extremely low because it corresponded to four measurements of methylene blue concentration obtained after each photocatalytic experiment. The higher errors observed for the methylene blue direct photolysis could be explained since they were calculated taking, into account, two duplicate experiments.

The results obtained proved the positive effect of using water as a matrix instead of using ethanol during the sol-gel process, as well as using lower temperature values for achieving high photocatalytic performance, leading to a more sustainable production process (in terms of solvent consumption and energy costs).

### 2.2. Morphology Characterization

**Figure 5:**
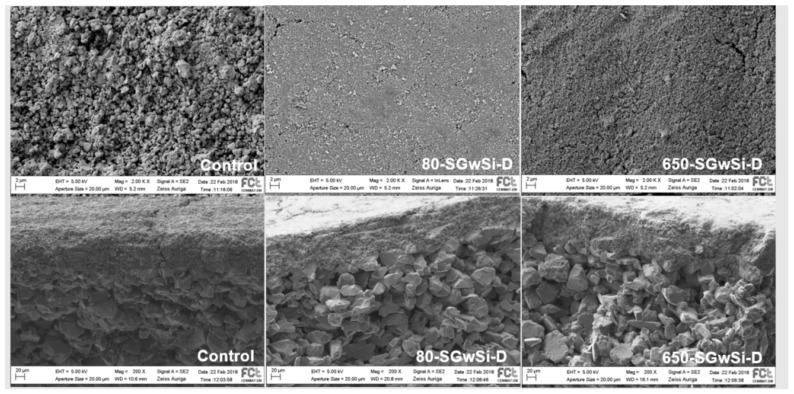
Top view (above) and cross-section (below) of the SEM images obtained for the control membranes, as well as for the solvent-free modified 80-SGwSi-D and 650-SGwSi-D membranes (magnification ×2000).

The morphology and homogeneity of the top layer and cross-section of the unmodified (control) and modified membranes using the solvent-free method were observed by SEM (Figure 5).

Figure 5 shows that the top layer of the control membrane presented an irregular surface with variable thickness. The 80-SGwSi-D and 650-SGwSi-D membranes exhibited a high nanoporous structure with a few surface irregularities due to the grains of titanium dioxide [19] and regular thickness. The 80-SGwSi-D membrane has a more uniform surface compared to the 650-SGwSi-D membrane that presented micro-cracks probably as a consequence of shrinkage during the final thermal protocol applied [23].

The cross-section images of the substrates presented in Figure 5 for the modified membrane showed that there was also no infiltration of the sol-gel solution through the membranes, showing a thickness with an average of 6.86 ± 1.04 µm for the 80-SGwSi-D and a reduction in the thickness of 5.60 ± 1.00 µm for the 650-SGwSi-D membrane, which could be explained due to the thermal densification of the film [24].

#### Porosity of Membranes

The porous characteristics of the membranes (80-SGwSi-D, 650-SGwSi-D, and control) were estimated in two different zones observed (Z_1_ and Z_2_) after image analysis with the software ImageJ (Table 3). The zone selection was random. Two zones were analyzed to check if the membrane morphology changes after the modifications were consistent.

The lower mean pore area obtained for the modified membranes (80-SGwSi-D and 650-SGwSi-D; 0.01 µm^2^) compared to the unmodified membrane (control; 0.05 µm^2^) might be indicative of a lower molecular weight cut off and might consequently lead to a higher pollutant rejection of the modified membranes (Table 3). Higher circularity values were determined for the modified membranes compared to the control, showing that the pores obtained were closer to perfect circles. The error bars associated with this parameter were also lower, showing a higher homogeneity for the modified 80-SGwSi-D and 650-SGwSi-D membranes compared to the control membrane.

Compared with the unmodified membrane (control), the modified membranes (80-SGwSi-D and 650-SGwSi-D) showed a much higher pore density (Table 3).

The average percent of porosity was slightly higher for the 80-SGwSi-D membrane (10.3%) compared to the control membrane (9.1%).

Feret’s diameters of 0.32–0.33 μm were observed for the control membrane, and, as expected, lower values of 0.12–0.15µm were observed for the two modified membranes (Table 3).

The modified membrane subject to 80 °C (80-SGwSi-D) had a higher homogeneity (since a higher similarity was observed in the estimated parameters in two different zones), showing that this was the best modification to achieve a reproducible narrow pore size distribution and symmetry of pores.

### 2.3. Contact Angle

The hydrophilic properties of the membranes tested in terms of photocatalytic activity were determined by measuring the water contact angle using the sessile drop method.

Higher hydrophilicity for 80-SGwSi-D and 650-SGwSi-D membranes was obtained as expected due to the increase in OH groups presence induced by UV-Light [25], exhibiting values for the first contact angle of 11 and 14, respectively, whereas the control membrane achieved a first average contact angle of 34 degrees (Figure S2). Comparing the contact angle obtained for 80-SGwSi-D with a modified membrane reported in a previous study using ethanol as a solvent (named SiO_2_-TiO_2_ L3) [19], the average contact angle obtained was similar (11 degrees), and thus the use of a free solvent process was also beneficial for achieving high hydrophilic membranes and, potentially, lower fouling tendency.

### 2.4. Membrane Filtration Performance

The hydraulic permeability of the control and the solvent-free 80-SGwSi-D and 650-SGwSi-D membranes were measured using a dead-end filtration system previously described [19]. The control membrane presented a higher hydraulic permeability (20,360 ± 3583 Lh^−1^m^−2^bar^−1^) when compared with the modified membranes (the membrane 80-SGwSi-D presented a hydraulic permeability of 4591 ± 371 Lh^−1^m^−2^bar^−1^, and the membrane 650-SGwSi-D presented a hydraulic permeability of 1864 ± 472 Lh^−1^m^−2^bar^−1^), which is a consequence of the higher pore size of the control membrane (Table 3). Table 4 shows the permeability for methylene blue during the filtration tests for the control and the solvent-free 80-SGwSi-D and 650-SGwSi-D membranes, measured in the absence and presence of UV radiation.

**Figure 6:**
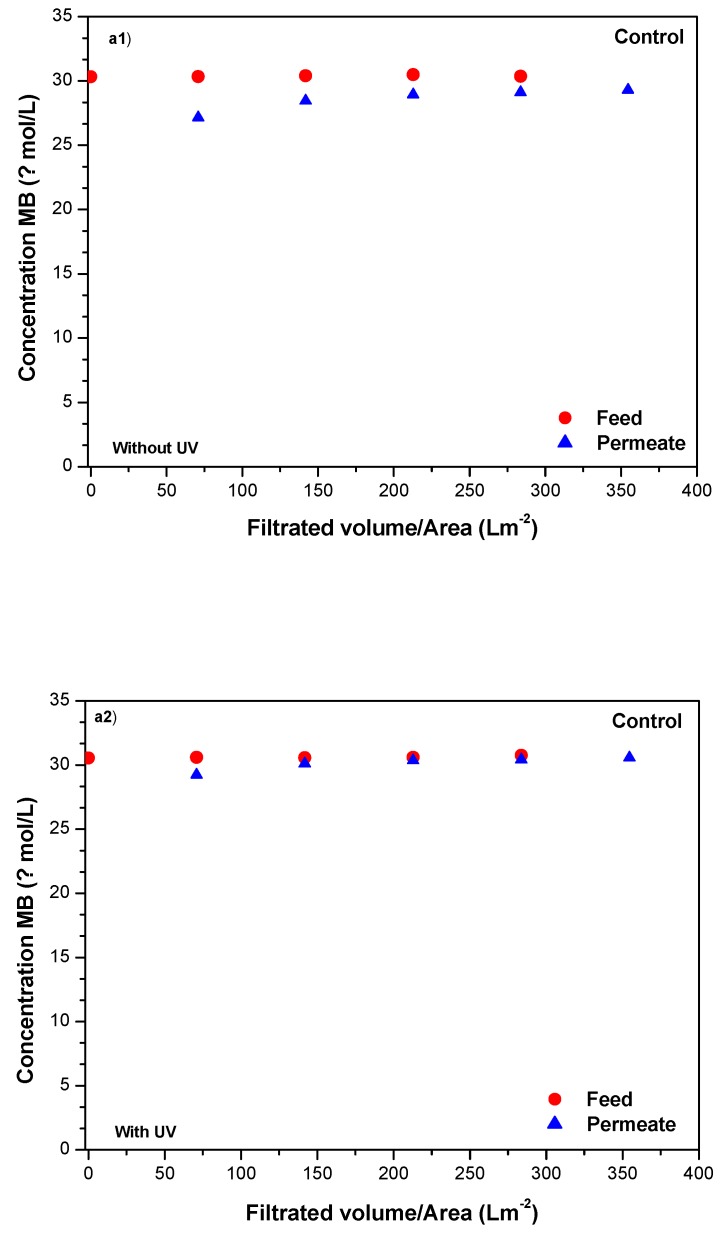

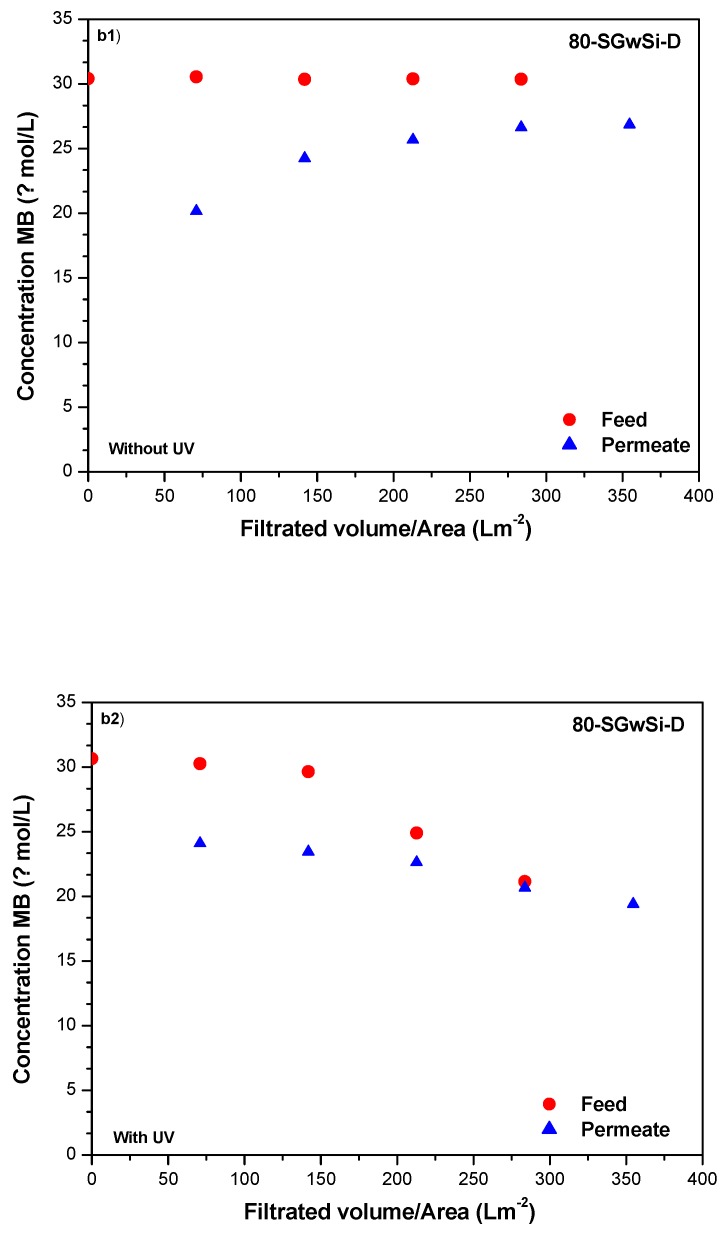

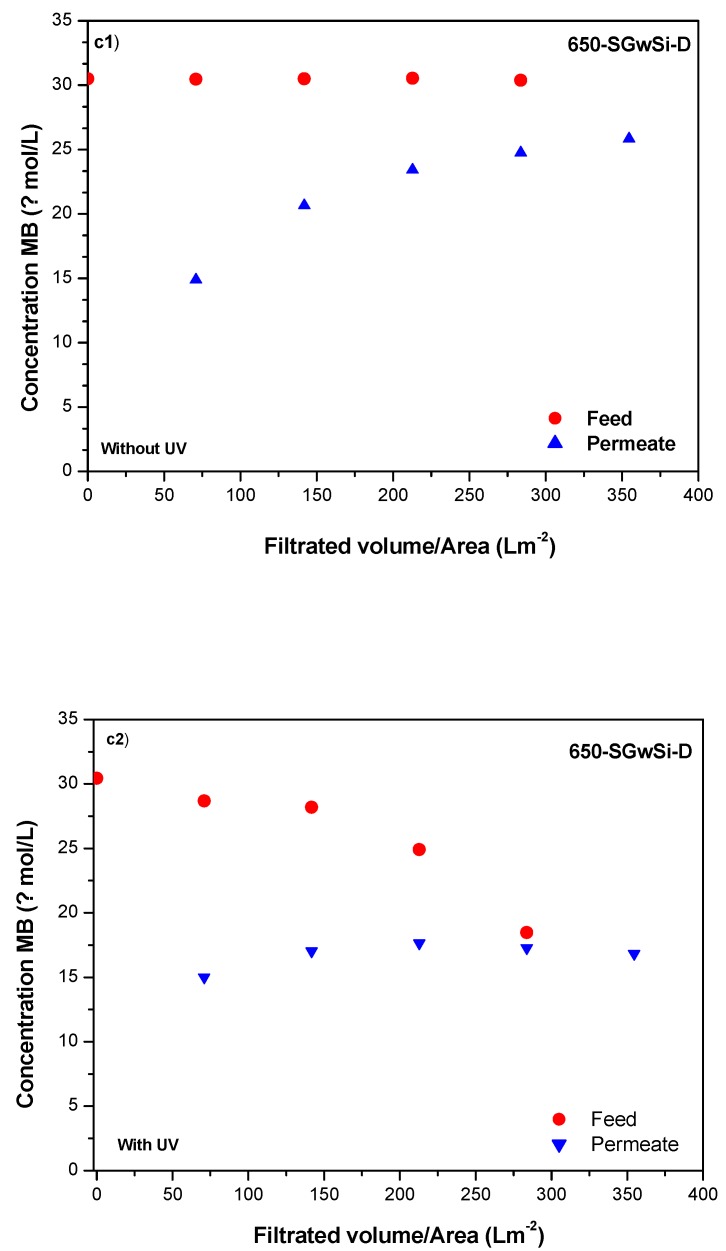
Comparison of filtration performance measured in terms of methylene blue (MB) concentration in feed and permeate samples with and without photolysis for the (**a**) control membrane, (**b**) modified 80-SGwSi-D membrane, and (**c**) the 650-SGwSi-D membrane.

Figure 6 represents the evolution of methylene blue degradation in the feed and permeate streams as a function of the volume filtrated normalized by the membrane area that will allow the comparison of these results with others obtained using different membrane areas.

The results obtained in Figure 6 showed that the control membrane could not retain methylene blue despite the initial small decrease in the concentration measured in the permeate due to adsorption. This result was expected, given the small molecular weight of methylene blue, and concurred with a result previously reported [19]. For the 80-SGwSi-D and 650-SGwSi-D membranes, in the absence of light, a more accentuated initial decrease of the methylene blue concentration was observed in the permeate as a consequence of adsorption, more pronounced for the 650-SGwSi-D membrane. This result showed that the reduction of the pore size of the modified membranes was translated into a higher adsorption capacity. Adsorption was the reason assumed for the decrease of the concentration of methylene blue in the permeate samples since, as with time, the concentration of methylene blue in the permeate increased, tending to the concentration of the feed (30 µM) in the assays conducted without UV.

The filtration tests conducted in the presence of UV light clearly showed the photocatalytic activity of both modified membranes. A clear decrease of methylene blue concentration was observed in the feed compartment in both tests due to its photodegradation, with a consequent downward trend of the concentration of methylene blue in the permeate in the case of the membrane 80-SGwSi-D, or achieving a constant concentration, in the case of the membrane 650-SGwSi-D.

Table 4 shows the significant improvement in the removal of methylene blue at the end of the filtration and UV experiments conducted with the modified membranes 80-SGwSi-D and 650-SGwSi-D to values of 37% and 45%, respectively. Taking into account that the feed vessel was completely stirred and the permeate flux constant, the estimated average exposure time of methylene blue was 19 min for both membranes (Table 4).

The chemical stability of the modified membranes (80-SGwSi-D and 650-SGwSi-D) were also tested to ensure that the TiO_2_ coating was not released from the substrate, after cleaning with distilled water and acid and basic solutions [4]. The results obtained by inductively coupled plasma-atomic emission spectroscopy are presented in Table 5.

Results showed that the concentration of titanium in the solutions after the membrane cleaning experiments were similar to the concentrations measured in the control solutions, confirming the chemical stability of the modified membranes.

Given their characterization results (Table 3) and the results obtained in terms of photocatalytic performance (Figure 3 and Figure 4), these membranes might prove effective in retaining microorganisms (such as bacteria and fungi) and inactivating them. Work is ongoing to prove these applications. As an example, when photocatalytic ceramic membranes, produced using this solvent-free method, were tested to treat surface water spiked with *Aspergillus fumigatus*, the results obtained showed high percentages of adsorption and retention of the spores for all treatments and that UV photolysis assured an effective treatment of the retentate [26]. In addition, the membranes produced in this work (without using solvents) proved to have a similar morphology and filtration performance when compared to photocatalytic membranes produced using solvents [19]. Since the photocatalytic membranes produced using solvents proved to be effective to treat wastewaters generated by the olive oil industry [20], the membranes detailed in this work are also expected to be effective to achieve high removals of total suspended solids, chemical oxygen demand, total organic carbon, and phenolic and volatile compounds.

### 2.5. Economic Benefits of using Water Instead of Solvents

The aim of this study was to adapt the production of SiO_2_-TiO_2_ sol-gel photocatalysts to modify commercial ceramic membranes rendering low cost and high productive photocatalytic materials for water decontamination.

Using water instead of an organic solvent in an industrial process brings environmental and economic benefits. The savings of ethanol achieved with this new method to produce photocatalytic membranes were calculated. The photocatalytic membrane (produced with ethanol) was already tested with real olive mill wastewaters with extremely promising results [20]. In this work, an average flux of 10 L/(m^2^ h) was obtained at 0.2 bar with a membrane with 0.029 m^2^ of area.

Thus, a membrane area of 82 m^2^ would be needed to treat a total annual volume of 1000 m^3^ (7 m^3^/day during five months, working 8 h/day) [27]. Given this, 1417 L of ethanol could be saved if these new solvent-free photocatalytic membranes were used. Thinking in economic terms, this represents a saving of 990 €, considering an average cost of 0.7 €/L of industrial ethanol and 1.55 €/m^3^ of industrial water in Portugal.

## 3. Materials and Methods

In the present study, commercial flat sheet silicon carbide membranes were provided by LiqTech International and used as substrates. The chosen reagent for sol-gel preparation of the SiO_2_ precursor was tetraethyl orthosilicate (TEOS) (98%; Sigma–Aldrich, St Louis, MO, USA). Degussa P25 titanium dioxide with 30–90 nm of nominal diameter provided by Evonik was used. All solvents employed in the sol-gel process were reagent-grade and used without further purification. Methylene blue (Merck, Darmstadt, Germany) was chosen to test the photocatalytic effectiveness of the modified membranes [28], and distilled water was used to measure the contact angle of the unmodified (control) and modified surfaces.

### 3.1. Modification of Ceramic Membranes

Silicon carbide flat membranes were cut (squares with an area of 11.4 cm^2^ used in the assays to test the photocatalytic performance detailed in Section 3.2.1, and circles with an area of 17.3 cm^2^ used in the membrane filtration tests detailed in Section 3.2.4; thickness of 0.3 cm), thoroughly cleaned with a 2% solution of MicroClean 90^®^, rinsed with distilled water, and heated at 80 °C overnight. Drop-casting was used as a deposition method. A previous modification strategy that proved to be effective in terms of photocatalytic activity, but employed solvents, served as the basis for this work [19]. The strategy was to use silicon carbide substrates and modify them using TEOS as silicon dioxide source, combined with Degussa nanoparticles as titania source. The optimized procedure was tested following a solvent-free sol-gel procedure conducted at room temperature conditions.

#### 3.1.1. Temperature Effect on Photocatalytic Activity

**Figure 7:**
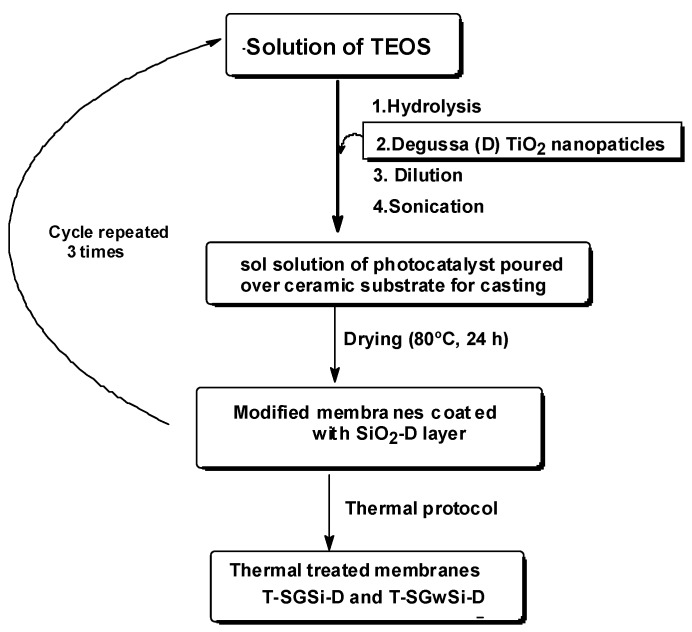
Representation of the modification process and designation of the membranes produced. T denotes the temperature applied over modified substrates samples and w differentiates the samples prepared using solvent free sol-gel process.

The membranes were coated with three layers of silicon dioxide combined with titanium dioxide Degussa (D) P25 nanoparticles (Figure 7), as detailed in previous work using ethanol as a solvent under acid-catalyzed sol-gel synthesis [19]. To test the influence of the final thermal protocol on the photocatalytic behavior of the membranes, the modified membranes were subject (at ambient atmosphere) to ramps of slow temperature increase (3 °C/min), up to 300 °C, 500 °C, and 650 °C. These temperatures were selected since the range 500 °C to 600 °C has been described as optimal for achieving good final photocatalytic properties in SiO_2_-TiO_2_ compositions [29] while ensuring the thermal stability of the commercial silicon carbide substrate. High temperatures (e.g., above 800 °C) may induce thermal stress by inducing defects at the SiO_2_/SiC interface [30]. The selected temperatures (300 °C, 500 °C, and 650 °C) were maintained for three hours, and the samples were then cooled down naturally. The membranes obtained were labelled as T-SGSi-D, where T denotes the temperature applied.

#### 3.1.2. Solvent-Free Process for the Production of Photocatalytic Membranes

The silicon carbide substrates were modified using the sol-gel process described above [19] but employing an aqueous matrix, maintaining the concentration of the acid catalyst, TEOS, and Degussa nanoparticles (Figure 7).

To avoid using ethanol, in this work, a concentrated stock solution of TEOS (1.69 M) was prepared in aqueous acidic conditions (pH = 1). The hydrolysis and condensation reactions to convert the silicon alkoxide TEOS, completely to SiO_2_, was guaranteed because two moles of water are needed for each mole of Si precursor. So, an excess of water can be used [31]. TEOS reagent is not miscible in water, so it is typically mixed with ethanol to improve its dispersion in water [32]. However, in this work, the miscibility was attained after 20 min due to the addition of acid as catalyst. The hydrolysis was maintained during 2 h, and the transparent stock solution was stable for one month. The hydrolyzed TEOS was diluted with distilled water and added to Degussa nanoparticles, with a final concentration of 0.045 M SiO_2_ and 0.050 M TiO_2_ that corresponds to 0.9:1 molar ratio of SiO_2_:TiO_2_ [16,19]. One milliliter of this sonicated sol-solution was deposited over silicon carbide membranes (11.4 cm^2^) by drop-casting and heated at 80 °C for 24 h to promote the condensation and form the SiO_2_ network. This protocol was repeated three times. A weight of 12 mg TiO_2_ was, therefore, coated in each membrane piece with 11.4 cm^2^ (1 mg/cm^2^). For the circular membranes used in the filtration tests, a weight of 18.3 mg TiO_2_ was coated in each membrane piece with 17.3 cm^2^ to achieve the same value of weight per unit area of 1 mg/cm^2^.

The membranes modified by this procedure were compared to membranes subjected to an additional thermal protocol: ramp of 3 °C/min up to a maximum temperature of 650 °C maintained during 3 h (at ambient atmosphere) and cooled down naturally.

The modified membranes obtained were identified as T-SGwSi-D, where T denotes the temperature applied over modified substrates samples, and w differentiates the samples prepared using water as a matrix in the sol-gel process.

### 3.2. Assessment of the Photocatalytic Membranes and Selection of the Most Promising One

#### 3.2.1. Assays to Test the Photocatalytic Performance

The evaluation of the photocatalytic activity of the modified membranes was performed, as previously described [19]. Methylene blue was employed as a tester dye to test the photocatalytic efficiency of the unmodified (control) and modified membranes [28]. A UV collimated beam set-up with an UVH-lamp type Z (UV-Technik meyer GmbH, Ortenberg, Germany) that emits polychromatic light, housed in a shuttered box with PN310 quartz (UV-Technik), was used. A calibrated radiometer (IL393, International Light, Newburyport, MA, USA), placed at the same height of the solution level in the Petri dish was used, to measure the maximum irradiance value (25 mW/cm^2^).

Double-walled glass Petri dishes (maintained at 23 ± 2 °C by the circulation of cold water) were placed beneath the UV source with 30 mL of a constantly stirred aqueous solution of 30 µM of methylene blue (pH 6.3) with and without a membrane (to test the direct photolysis effect). The concentration of methylene blue was measured in the different samples taken at different experimental times (0, 20, 40, and 60 min) and quantified based on a calibration curve performed after measuring the absorbance measurements at 664 nm using a UV-Vis spectrophotometer (Ultrospec 2100 pro-UV-VIS, Biochrom Ltd., Cambridge, UK). Four absorbance measurements were taken in each sample. To test the adsorption capacity of the unmodified and modified membranes, dark reactions were performed under the same conditions. The percent removal obtained after 60 min was calculated using Equation (2):

(2) % removal of methylene blue=[(C0−C60)C0]×100

where C_0_ is the concentration of the methylene blue measured at time zero and C_60_ the concentration measured after 60 min of UV exposure.

For the most promising membranes, five consecutive degradation experiments were conducted under the same conditions using two different batches of membranes modified to ensure the photocatalytic results obtained were reproducible and that the membranes could be reused.

A last long-term assay was conducted with the most promising membranes to follow the degradation of methylene blue.

Membrane characterization (in terms of morphology and contact angle) was performed after membrane evaluation in terms of their photocatalytic activity.

#### 3.2.2. Morphology Characterization

Scanning electron microscopy (SEM) was used to characterize the top surface and cross-section of the most promising modified membranes and compare it with the unmodified substrate after coating with an Au/Pd thin film (15 nm). They were analyzed using a Carl Zeiss AURIGA CrossBeam Workstation instrument equipped with an Oxford Energy Dispersive X-ray Spectrometer (EDS). The different top surfaces were analyzed using the ImageJ software (an open source image processing program, University of Wisconsin, Madison, WI, USA) [33,34,35]. Two random zones were analyzed to check if the membrane morphology was consistent.

#### 3.2.3. Contact Angle

Three different places were randomly chosen in each membrane to measure the contact angle of a sessile drop of distilled water (10–12 µL) using a KSV CAM2008 equipment. Twenty frames were attained for each measurement with a frame interval of 100 ms.

#### 3.2.4. Membrane Filtration Assays

A dead-end filtration system coupled to a vacuum pump (model: DOA-P504A-BN, GAST Manufacturing) was placed under the UV system to compare the performance of the different solvent-free modified membranes (80-SGwSi-D and 650-SGwSi-D) produced in this work and the control. A total of 250 mL of a 30 µM methylene blue solution was filtered by the 4.7 cm diameter membranes with a 0.2 bar transmembrane pressure using this setup. Control assays were conducted in the absence of UV light. The total removal of methylene blue that includes the effect of membrane filtration (rejection and adsorption) and photolysis (direct and indirect photodegradation) was calculated using Equation (3):

(3) % total removal of methylene blue=[(Cfeed−Cpermeate)Cfeed]×100

The photocatalytic degradation of methylene blue in the feed compartment was calculated through Equation (4):

(4) % photocatalytic degradation of methylene blue=(Cfeedt−Cfeedt−1)Cfeedt

After the filtration experiments, different cleaning protocols, described by Fraga et al. [4] in pilot-scale experiments conducted with tubular SiC unmodified membranes, were tested to assess the chemical resistance of the modified membranes used in the experiments described above by monitoring the possible release of nanomaterials from the membranes. The cleaning protocol consisted of rinsing the membrane with 100 mL of water, followed by chemical cleanings using 100 mL of NaOH 4% (*w*/*v*) and citric acid 2% (*w*/*v*) solutions. All the steps were performed at 65 ± 5 °C [4]. The Ti element was analyzed by inductively coupled plasma-atomic emission spectroscopy (ICP-AES) (Horiba Jobin-Yvon, Longjumeau, France).

## 4. Conclusions

A new solvent-free sol-gel process was proposed to produce photocatalytic membranes that could be reused and maintain their photocatalytic activity. The modification process proposed was low cost and environmentally friendly (without the use of solvents and involving a low-temperature thermal treatment). These membranes proved to be effective and might be promising for the removal and inactivation of microorganisms from wastewater. Therefore, these membranes should be further tested in pilot-scale hybrid reactors that combine membrane filtration, UV, and advanced oxidation processes.

## Figures and Tables

**Table 1:** Percent removal and adsorption measured using the unmodified (control) and modified membranes (60 min).

Membranes	Methylene Blue Removal (%)	Methylene Blue Adsorption (%)
**Control**	48	13
**80-SGSi-D**	72	20
**300-SGSi-D**	77	27
**500-SGSi-D**	73	29
**650-SGSi-D**	82	31
**80-SGwSi-D**	77	17
**650-SGwSi-D**	74	24

**Table 2:** Calculated degradation rate constants (k) and half-life time (t_1/2_) values.

Membranes	k (min^−1^)	t_1/2_ (min)
**Control**	0.0110	63
**Methylene blue**	0.0040	192
**80-SGSi-D**	0.0220	31
**300-SGSi-D**	0.0244	28
**500-SGSi-D**	0.0216	32
**650-SGSi-D**	0.0285	24
**80-SGwSi-D**	0.0275	25
**650-SGwSi-D**	0.0225	31

**Table 3:** Porous characterization of the membranes (magnification of x 2000).

Membranes *	Control Z_1_ × 2000	Control Z_2_ × 2000	80-SGwSi-D Z_1_ × 2000	80-SGwSi-D Z_2_ × 2000	650-SGwSi-D Z_1_ × 2000	650-SGwSi-D Z_2_ × 2000
**Pore density (µm^−2^)**	1.7	1.5	11.6	11.1	3.8	10.1
**Mean pore area (µm^2^)**	0.05 ± 0.16	0.06 ± 0.23	0.01 ± 0.02	0.01 ± 0.03	0.01 ± 0.03	0.01 ± 0.01
**Minimum pore area (µm^2^)**	0.003	0.003	0.003	0.003	0.003	0.003
**Maximum pore area (µm^2^)**	3.409	7.136	0.79	1.00	2.15	0.19
**Porosity (%)**	9.0	9.3	12.0	8.6	3.4	6.3
**Average circularity**	0.77 ± 0.39	0.80 ± 0.26	0.90 ± 0.19	0.93 ± 0.16	0.93 ± 0.16	0.94 ± 0.15
**Average feret diameter (µm)**	0.32 ± 0.26	0.33 ± 0.45	0.15 ± 0.12	0.13 ± 0.11	0.13 ± 0.13	0.12 ± 0.07
**Maximum feret’s diameter (µm)**	1.000	6.963	3.215	2.896	5.461	1.079
**Minimum feret’s diameter (µm)**	0.073	0.083	0.081	0.078	0.076	0.079

* two different zones analyzed (Z_1_ and Z_2_).

**Table 4:** Permeability, as well as methylene blue photocatalytic degradation and total removal during membrane filtration (MF), conducted with and without photolysis (UV).

	Control		80-SGwSi-D		650-SGwSi-D	
	MF	MF + UV	MF	MF + UV	MF	MF + UV
**MB permeability (Lh^−1^m^−2^bar^−1^) during the test**	19,126 ± 950	19,857 ± 3490	3479 ± 280	2645 ± 705	1955 ± 135	2486 ± 363
**Time of filtration (min)**	5	5	30	38	50	37
**% Photocatalytic degradation in the feed**	n.a	0	n.a	31	n.a	39
**% Total removal**	3	0	12	37	15	45

**Table 5:** The concentration of titanium measured in control solutions and after filtration with three different cleaning solutions at 65 ± 5 °C (distilled water, citric acid 2% (*w*/*v*) and NaOH 4% (*w*/*v*) using the control membrane and the modified 80-SGwSi-D and 650-SGwSi-D membranes.

	Samples	Ti (mg/L)
**Control solutions, no filtration performed**	Distilled water	<0.005
	Citric acid 2% (*w*/*v*)	0.05
	NaOH 4% (*w*/*v*)	0.05
**Control membrane**	Distilled water	0.03
	Citric acid 2% (*w*/*v*)	0.04
	NaOH 4% (*w*/*v*)	0.09
**80-SGwSi-D membrane**	Distilled water	<0.005
	Citric acid 2% (*w*/*v*)	0.05
	NaOH 4% (*w*/*v*)	0.05
**650-SGwSi-D membrane**	Distilled water	<0.005
	Citric acid 2% (*w*/*v*)	0.05
	NaOH 4% (*w*/*v*)	0.12

## Supplementary Materials

The following are available online, Figure S1: Pseudo-first-order kinetics for the removal of methylene blue for membranes tested; Figure S2: Comparison of the time course of water contact angle for control, 80-SGwSi-D, and 650-SGwSi-D modified substrates.
