# Supplementary material for: Solvent-Free Process for the Development of Photocatalytic Membranes
Source: Molecules. 2019 Dec 6;24(24):4481. doi: 10.3390/molecules24244481 (PMC6943574; doi:10.3390/molecules24244481)
Supplement: Supplementary file 1 [file molecules-24-04481-s001.pdf]

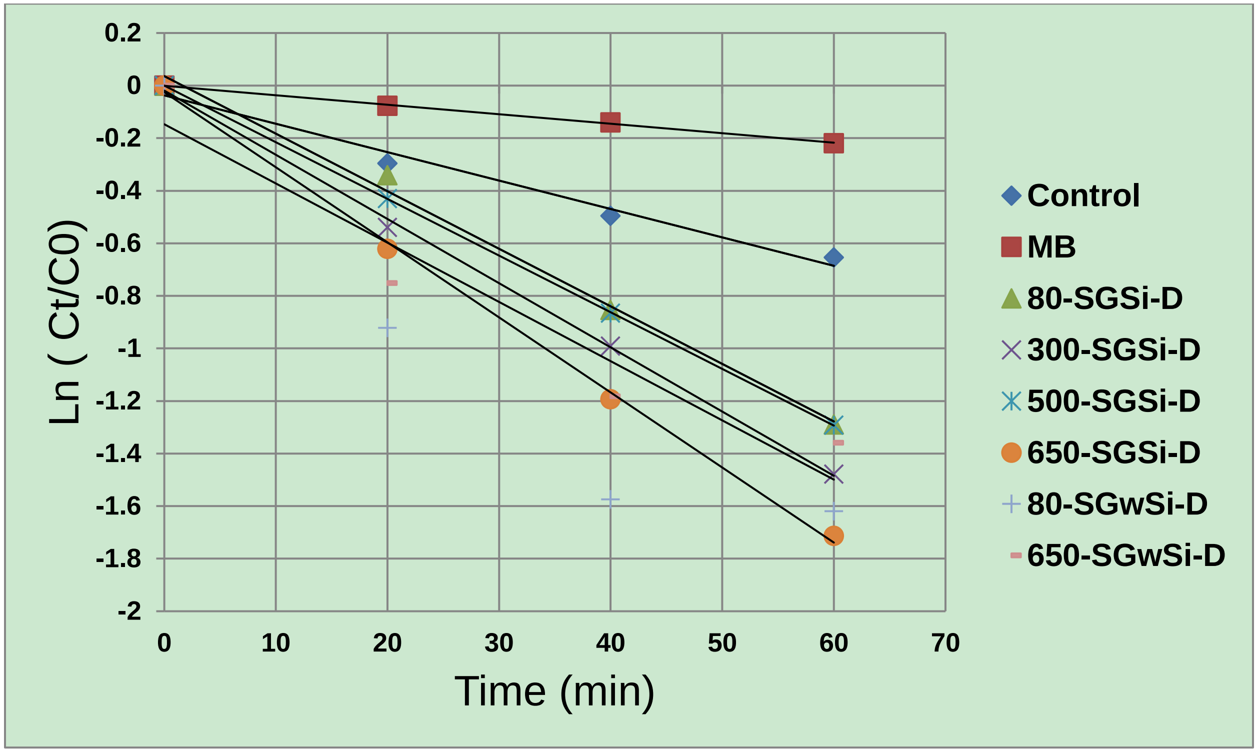

Fig. S1. Pseudo-first-order kinetics for the removal of methylene blue for membranes tested.

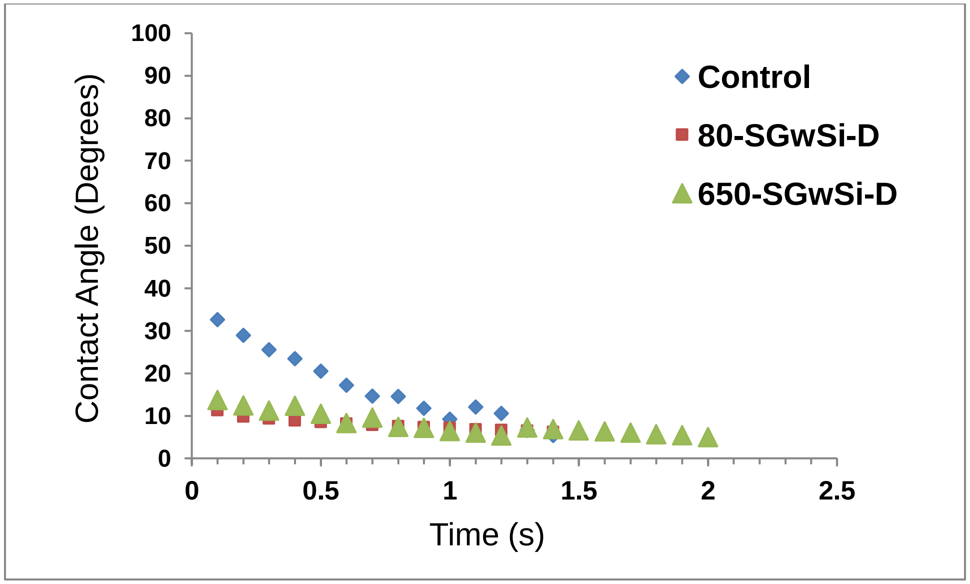

Fig. S2 Comparison of the time course of water contact angle for **Control**, **80-SGwSi-D** and **650-SGwSi-D** modified substrates.
